# Effects of Xinjiang wild cherry plum (*Prunus divaricata* Ledeb) anthocyanin-rich extract on the plasma metabolome of atherosclerotic apoE-deficient mice fed a high-fat diet

**DOI:** 10.3389/fnut.2022.923699

**Published:** 2022-07-25

**Authors:** Jing Shen, Xing Li, Xin Zhang, Zhen Li, Gulisitan Abulaiti, Yang Liu, Jun Yao, Pei Zhang

**Affiliations:** ^1^College of Pharmacy, Xinjiang Medical University, Urumqi, China; ^2^Department of Pharmacy, Fifth Affiliated Hospital of Xinjiang Medical University, Urumqi, China; ^3^School of Basic Medical Sciences, Shaoyang University, Shaoyang, China

**Keywords:** *Prunus divaricata* Ledeb, anthocyanin, plasma, metabolomics, atherosclerosis

## Abstract

It is well-known that many vegetables and fruits have abundant polyphenols, such as anthocyanins, which benefit many cardiovascular diseases due to their anti-oxidative and anti-inflammatory effects. To explore the protective effect of anthocyanin on atherosclerosis from a metabolic perspective, alterations in plasma metabolic profiling of apoE-deficient (apoE^–/–^) mice in response to treatment with anthocyanin extracts derived from Xinjiang wild cherry plum (*Prunus divaricata* Ledeb) peel was investigated through UHPLC-Q-TOF/MS. The mice were fed with a normal diet or high-fat diet supplementation with or without anthocyanin extracts (ACNE, 75, 150, 250 mg/kg body weight) for 18 weeks, corresponding to control (Con), model (Mod), and treatment group (LD, low dose; MD, medium dose; HD, high dose), respectively, along with a positive control group (posCon, treatment with Atorvastatin, 0.003 mg/kg body weight). The results showed that ACNE could significantly enhance the antioxidant capacity and lower the plasma lipid, but have no evident influence on the body weight of apoE^–/–^ mice. A series of differential metabolites, predominantly related to lipid metabolism, were identified, including docosahexaenoic acid, palmitoyl ethanolamide, stearoylcarnitine, L-palmitoylcarnitine, indoxyl sulfate (IS), 1-palmitoyl-lysophosphatidylcholine, phenylacetylglycine (PAGly), and so on. Among these, both IS and PAGly were host-microbial metabolites. These differential metabolites were mainly enriched in the pathway of glycerophospholipid metabolism and linoleic acid metabolism. Several important enzymes related to glycerophospholipid metabolisms such as LCAT, LPCAT, GPCPD1, PLA2G1B, PPARG, LIPE, PNPLA2, AGPAT1, and ENPP2 were recognized as underlying targets for anti-atherogenic effects of ACNE. These findings suggest that ACNE derived from Xinjiang wild cherry plum exhibits protective effects against atherosclerosis via modulating glycerophospholipid metabolism.

## Introduction

Atherosclerosis is a diffuse, chronic inflammatory, immunometabolic, vascular disease, which can cause three clinical diseases, including coronary heart disease, ischemic stroke, and peripheral vascular disease, due to luminal narrowing or thrombi deposition resulting in obstructing blood flow to the heart, brain, or lower extremities (1). Among these sequelae, ischemic heart disease and stroke account for 85% of cardiovascular deaths and 28% of all-cause mortality (2). As a lipoprotein-driven disease, atherosclerosis is characterized by the formation of complex atherosclerotic plaques at specific sites of the arterial tree, which experience intimal inflammation, necrosis, fibrosis, and calcification (1). Overall, the progression of atherosclerosis involves lipoprotein metabolism, oxidative stress, inflammation, and immunometabolism, including a series of sequential events, LDL deposition, endothelial dysfunction, subendothelial accumulation of foam cells (macrophages with oxidized LDL), and formation of fatty streaks, under the activation of pro-inflammatory factors (3). Previous studies have demonstrated that several cardiovascular disease (CVD) risk factors, such as hypertension, diabetes mellitus, dyslipidemia, and cigarette smoking, have been closely linked with the atherosclerotic process. To date, risk factors and targeted therapies for CVD can be appropriately evaluated with the help of many diagnostic techniques, both invasive (such as selective coronary arteriography) and non-invasive (such as blood biomarkers, stress testing, CT, and nuclear scanning). Various medications for slowing the progression of atherosclerosis have been developed, encompassing hyperlipidemia medications, anti-platelet drugs, hypertension, and hyperglycemia medications (4). Among these therapies, lowering LDL-C by statin remains the cornerstone for the medical prevention and treatment of atherosclerotic disease because of its efficacy and safety. However, there are still considerable challenges in the treatment of atherosclerosis, such as incompletely retarding the progression of atherosclerosis, suboptimal treatment adherence due to adverse effects, and clinician inertia (5, 6). Thereby, it is needed to develop novel therapies, natural alternatives, or diet therapy for the prevention and treatment of atherosclerosis.

Wild cherry plum (*Prunus divaricata* Ledeb) belongs to *the Prunus* genus, *Prunoideae* (or *Amygdaloideae*) subfamily of the *Rosaceae* family. It mainly naturally occurs in temperate regions of the northern hemisphere, including North America, Europe, Caucasia, and Central Asia. In China, the population of *Prunus divaricata* Ledeb has been found in the Ili River Valley of the Western Tianshan Mountains, located at Huocheng County, Ili Kazakh Autonomous Prefecture of Xinjiang Uygur Autonomous Region (7). The fruit of *Prunus divaricata* Ledeb is a vital wild fruit for human beings and other wild animals, usually consumed fresh or processed as juice, sauce, or jam by people. This plum has high nutritional value as it possesses multiple beneficial components, such as polyphenols, flavonoids, polysaccharides, alimentary fiber, ascorbic acid, and minerals (8, 9). One of the most abundant compositions is a class of anthocyanins, which has various bioactivities and health benefits, such as free radical scavenging, antioxidant, anti-inflammatory, antimicrobial, and anticancer activity (10). Anthocyanins, as a subclass of flavonoids, are water-soluble pigments, which widely occur in flowers, fruits, and leaves of a plethora of plants and vegetables, and are responsible for brilliant colors of pink, red, blue, and purple (10). Amounts of studies and epidemiological investigations have suggested a robust association existing between high consumption of fruits and vegetables and a lower mortality rate (11, 12), probably attributed to polyphenols, especially anthocyanins. The beneficial effects of anthocyanins are mainly due to their antioxidant capacity stemming from multiple phenolic hydroxyl groups attached to their ring structure. Their anti-oxidative effects and capacity of inducing the expression of relevant enzymes confer the ability to reduce oxidative stress and inflammatory injuries that can ameliorate the progression of atherosclerosis (13). Additionally, it has been reported that many cardiovascular health benefits such as lower lipids and lower arterial stiffness have been available through the intake of anthocyanins (12). Together, anthocyanins exert anti-inflammatory, antioxidant, and antithrombotic activities via multiple complex mechanisms of action, such as regulation of gene and miRNA expression, and cell-signaling pathways (14). Despite great advances in molecular mechanisms of anthocyanin action have been made, it is still far from entirely clear.

Metabolomics is a newly developed omics technology over the past two decades, with the main purpose of qualitative and quantitative analysis of all low molecular weight metabolites (<1,500 Da) in given samples. It has become a powerful tool for discovering potential biomarkers and exploring molecular mechanisms underlying diseases, with widespread application to nutrition and health (15). It has been reported that integrating analysis of metabolome and transcriptome was employed to reveal the association between color changes and anthocyanins (16). Recently, Chen et al. (17) investigated the effects of anthocyanin extracts from bilberry and purple potato on the plasma metabolomic profile of Zucker diabetic fatty rats. Their results suggested that two anthocyanin extracts had different effects on the plasma metabolic profile of diabetic rats. This prompts that the action of anthocyanin extracts existing across species differences might be because of different composition and abundance. Likewise, metabolomics and nutrigenomics have been used to explore the vascular benefits of blueberries (18). The results revealed that anthocyanin metabolites mediated the vascular bioactivities of blueberries and affected cellular gene expression. At present, to our best knowledge, the effects of anthocyanins extracted from Xinjiang wild cherry plum peel on atherosclerosis have not been reported. Here, we investigate metabolic changes of a plasma sample from apoE^–/–^ mice in response to anthocyanin extracts derived from Xinjiang wild cherry plum, with the aim to elucidate the action mechanism of anthocyanins.

## Materials and methods

### Anthocyanins extract

The anthocyanins extract (ACNE) from Xinjiang wild cherry plum (*Prunus divaricata* Ledeb) was prepared according to our previous approach (9). The details were provided in Supplementary Information. The purity of anthocyanins expressed as cyanidin 3-glucoside was determined as 48% by a pH-differential method as previously described (19). The composition of ACNE was analyzed by LC-MS/MS, mainly including cyanidin 3-galactoside, cyanidin 3-glucoside, cyanidin 3-rutinoside, and cyanidin 3-xyloside (9).

### Animals and feeding

All procedures with laboratory animals were approved by the Committee on Animal Research and Ethics of Xinjiang Medical University. All apoE^–/–^ mice (male 36, female 36) of 5–6 weeks of age were purchased from the Beijing Vital River Laboratory Animal Technology Co., Ltd. (Beijing, China) (SYXK [Jing] 2017-0033). Mice were maintained under standard husbandry conditions provided by the Laboratory Animal Center of Xinjiang Medical University (SYXK [Xin] 2018-0003) and were housed in colony cages with controlled temperature (20–24°C) and humidity (50–70%), as well as a 12-h light/dark cycle. They were supplied with *ad libitum* feeding of food and water.

After 1 week of accommodation, mice were randomly divided into six groups, including control (Con), model (Mod), low-, medium-, and high-dose (corresponding to LD, MD, HD) extract treatment (75, 150, 250 mg/kg body weight), and positive control (posCon, 0.003 mg atorvastatin/kg body weight) groups, with six mice in each group. Mice in control and other groups were fed with a normal diet and high-fat diet (containing 20% proteins, 50% carbohydrates, 21% fat, 0.15% cholesterol, permission No. [Su Feedstock]: 2018-0030, Medicience, Yangzhou, Jiangsu province, China) for 12 weeks, respectively. After 12 weeks, in each group except for the control group, one animal was randomly selected for sacrifice under pentobarbital-induced anesthesia and the development of atherosclerosis was assessed by the morphology of aortic arch, size of the plaque, and plasma lipids (TG, TC, LDL-C, and HDL-C). Subsequently, mice were intervened for 18 weeks. In LD, MD, and HD groups, mice were administrated by oral gavage with ACNE at doses of 75, 150, and 250 mg/kg body weight, respectively. In the posCon group, mice received atorvastatin (0.003 mg/kg body weight) in the same administration manner. In the Con and Mod groups, mice were treated with equivalent saline solution. During the total of 30-week feeding, the weekly feed intake and body weight were monitored. At the end of the experiment, after overnight fasting, mice were anesthetized by intraperitoneal injection of 2% sodium pentobarbital (0.2 ml/20 g body weight) for sacrifice. Plasma samples were collected by cardiac puncture and centrifuging the blood samples at 3,000 × g for 15 min at 4°C. The plasma samples were stored separately at −80^°^C until further analysis.

### Biochemical assays

The plasma levels of total triglycerides (TG), total cholesterol (TC), low-density lipoprotein cholesterol (LDL-C), high-density lipoprotein cholesterol (HDL-C), and activities of alanine aminotransferase (ALT) and aspartate aminotransferase (AST) were measured using an automatic ELISA analyzer (Rayto RT-6100) and commercial kits (A110-1-1, A111-1-1, A112-1-1, A113-1-1, C009-2-1, C010-2-1, Nanjing Jiancheng Institute of Bioengineering, Nanjing, China), following the manufacturer’s instructions. The plasma level of C-reactive protein (CRP) was assayed with an ELISA kit (E-EL-M0053C, Elabscience Biotechnology Co., Ltd., Wuhan, China). To assess the effects of ACNE on plasma antioxidant capacity, the activities of SOD, GPx, TAOC, and MDA were determined by using commercial kits purchased from Nanjing Jiancheng Institute of Bioengineering (Nanjing, China), according to the manufacturer’s instructions.

### Plasma metabolite extraction

For characterization of endogenous metabolic profiling, 400 μl of extraction solvent (acetonitrile:methanol = 1:1, v/v) containing the isotope-labeled internal standard mixture was added to 100 μl plasma. After 10 s of vortex, the sample was transferred to the ice-cold water bath and sonicated for 10 min. Then, the sample was placed in a −40^°^C fridge for 1 h, followed by centrifugation at 4^°^C, 13,000 × g for 15 min. About 400 μl of supernatant was collected and dried under vacuum. The dried extract was resuspended in 100 μl of 50% acetonitrile aqueous solution and vortexed for 30 s. After centrifugation at 4^°^C, 13,000 × g for 15 min, and 75 μl of supernatant was pipetted into LC insert in glass LC vial up to UHPLC-MS analysis. For quality control (QC) sample preparation, an aliquot of 10 μl of each plasma sample extract (supernatant) was pooled into one, and mixed together.

### UHPLC-QTOF-MS metabolomics data acquisition

The metabolomic analysis was performed on a UHPLC system (1290 Infinity LC, Agilent Technologies) coupled with a Triple TOF 6600 mass spectrometer (AB Sciex, Boston, MA, United States) in Shanghai Biotree Biotech Co., Ltd. The plasma metabolite extracts were separated on an ACQUITY BEH Amide column (2.1 × 100 mm, 1.7 μm, Waters, United States) at a flow rate of 0.5 ml/min. The mobile phases consisted of water containing 25 mmol/L ammonium acetate and 25 mmol/L ammonia (A) and acetonitrile (B). The analytes were eluted with a gradient program, listed as follows: 0.0–0.5 min, 95% B; 0.5–7.0 min, 95–65% B; 7.0–8.0 min, 65–40% B; 8.0–9.0 min, 40% B; 9.0–9.1 min, 40–95% B; and 9.1–12.0 min, 95% B. The column oven was set at 25^°^C and the sample injection volume was 2 μl. During the operation, all the samples (in the sample tray) were kept at 4^°^C. The analytes were detected with a mass spectrometer in both positive and negative ionization modes. The operation parameters of the ESI source were set as follows: ion source gas1 (Gas1): 60 psi, ion source gas2 (Gas2): 60 psi, curtain gas (CUR): 35 psi, source temperature (TEM): 600^°^C, ion spray voltage floating (ISVF): 5,000 V (Pos)/-4,000 V (Neg), TOF MS scan m/z range: 60–1,000 Da, product ion scan m/z range: 25–1,000 Da, TOF MS scan accumulation time: 0.2 s/spectra, and product ion scan accumulation time: 0.05 s/spectra. The MS/MS were acquired according to an information-dependent acquisition (IDA). During the data acquisition, the data acquisition and analysis software (Analyst TF 1.7, AB Sciex, Framingham, MA, United States) was used to evaluate the full-scan survey MS data based on preselected criteria. The IDA settings included declustering potential (DP) ± 60 V (positive and negative ionization mode), collision energy 35 ± 15 eV, exclude isotopes within 4 Da, and 6 candidate ions to monitor per cycle. Three analytical replications were done for all MS data.

### Metabolomics data processing and analysis

The raw LC-MS data were converted into mzXML files by ProteoWizard. Then, mzXML files were imported into R software (v4.0.5, 2021, R Foundation for Statistical Computing, Vienna, Austria) and several essential preprocessing steps, such as peak identification, peak matching, retention time correction, and peak integration were done by XCMS package. The generated three-dimensional data matrix consisting of the retention time values, mass to charge ratio (m/z) values, and peak intensities were further processed. The peaks with RSD% (calculating in the QC samples) > 30% were removed. Also, the peaks with missing values in more than 50% of samples were filtered out. For the left missing values, the data were filled up with half of the minimum value. After that, a complete data matrix was obtained. To tentatively recognize the identity of the peaks, the retention time, m/z, and MS/MS fragment ions were mapped to the library consisting of information from the online database HMDB,^1^ KEGG,^2^ PubChem,^3^ Metlin,^4^ and in-house MS2 library (Biotree, Shanghai) established with authentic standards. Subsequently, the data matrix was subjected to normalization based on the peak intensities of internal standards before multivariate analysis.

To screen the differential metabolites, the primary multivariate analyses, including principal component analysis (PCA) and orthogonal projections to latent structure-discriminant analysis (OPLS-DA), were performed by using SIMCA software (V16.0.2 Sartorius Stedim Data Analytics AB, Umeå, Sweden). Before building PCA and OPLS-DA models, the input data matrix was pretreated with log transformation and autoscaling (unit variance, UV). The quality of the OPLS-DA model was evaluated by a 7-fold cross-validation and permutation test. Meanwhile, univariate analysis (student’s *t*-test) was also carried out. The significantly changed metabolites were initially screened out by VIP > 1 and *p* < 0.05. To obtain more reliable differential metabolites, the preliminary differential metabolites were subjected to more stringent screening criteria, including MS2 score > 0.75, VIP > 1.5, and FC (fold change) > 1.5, with implementation by an in-house R script.

### Pathway enrichment and protein network analysis

To understand the origin and function of differential metabolites, metabolic pathway enrichment was performed by using the online tool MetaboAnalyst 5.0 (20). The pathway with *p* < 0.05 was considered an enriched pathway with statistical significance. To identify some key proteins linking the effects of ACNE to the significantly altered metabolites, the protein-protein interactions (PPIs) network between proteins associated with differential metabolites was built as previously described (21). In brief, the HMDB 5.0 (proteins, in XML format, Sep 24, 2021) was downloaded to local. Then, the metabolite-related proteins were extracted by using an in-house R script. The PPIs network was constructed by the online tool STRING (version 11.5), with Textmining and Experiments as active interaction sources. The network was analyzed and visualized with Cytoscape 3.8.0 for identifying hub proteins.

### Statistical analysis

The body weight and biochemical measures were statistically analyzed with one-way ANOVA followed by Bonferroni’s test using SPSS software (version 16, SPSS, Inc., Chicago IL). The results were presented as mean ± standard deviation (mean ± SD) and considered as statistical significance when *p* < 0.05. The column plots were generated by GraphPad Prism (version 7.00, GraphPad Software, Inc., La Jolla, CA).

Heatmap was used to represent the relative abundance of metabolites detected in each sample with color intensity. According to the differential metabolites data, the heatmap was generated by the function pheatmap in the R package pheatmap (version 1.0.12).

## Results

### Influence of anthocyanins extract on body weight

Body weight was regularly measured throughout the entire study. After 12-week feeding with a normal diet (Con group) or high-fat diet (Mod, LD, MD, HD, and posCon groups), the apoE^–/–^ mice were subjected to treatment with saline solution (Con, Mod group), ACNE (LD, MD, HD group), or positive drug (posCon group) through gavage for 18 weeks. As illustrated in Supplementary Figure 1A, there was no significant difference in body weight between the Mod and Con groups before and after treatment. Both apoE^–/–^ mice in the Mod and Con groups slowly obtained weight gain with increasing feeding time. Before treatment, the body weight of mice in the Mod, LD, MD, HD, and posCon groups was closely equal (Supplementary Figure 1B) to that of the Con group. After treatment for 6 weeks, ACNE had no significant influence on body weight compared to the Mod group, as same with atorvastatin. After 18 weeks of treatment, ACNE and atorvastatin slightly decreased the body weight when compared to the Mod group.

It was well-known that intake of high-fat feed could result in the accumulation of lipids, which was associated with chronic low-grade inflammation. The circulatory level of C-reactive protein (CRP) was determined by ELISA. The result showed that high-fat feeding significantly increased CRP in comparison to normal feeding, as seen in Supplementary Figure 1C. While, supplementation of ACNE or administration of atorvastatin (positive control drug) could reverse the increased level of CRP caused by high-fat feeding, especially for a high dose of ACNE (Supplementary Figure 1C).

### Antioxidant capacity of anthocyanin extracts

Polyphenols are an important class of compounds with antioxidant activity. Thus, the antioxidant capacity of plasma was measured. Compared to the Con group, the activities of SOD and GPx were significantly decreased in the Mod group, as well as the level of TAOC (Supplementary Figures 2A–C). Nevertheless, the level of MDA was significantly increased resulting from high-fat feeding (Supplementary Figure 2D). Supplementation of ACNE significantly increased the activities of SOD and GPx, along with the level of TAOC in a dose-dependent manner, compared to the Mod group. The level of MDA was significantly reduced by intervention of ACNE but without an evident dose-dependent fashion. The above results suggested that ACNE enhanced the antioxidant capacity of plasma. Likewise, atorvastatin elevated plasma antioxidant levels.

### Impact of anthocyanins extract on plasma lipids

**FIGURE 1:**
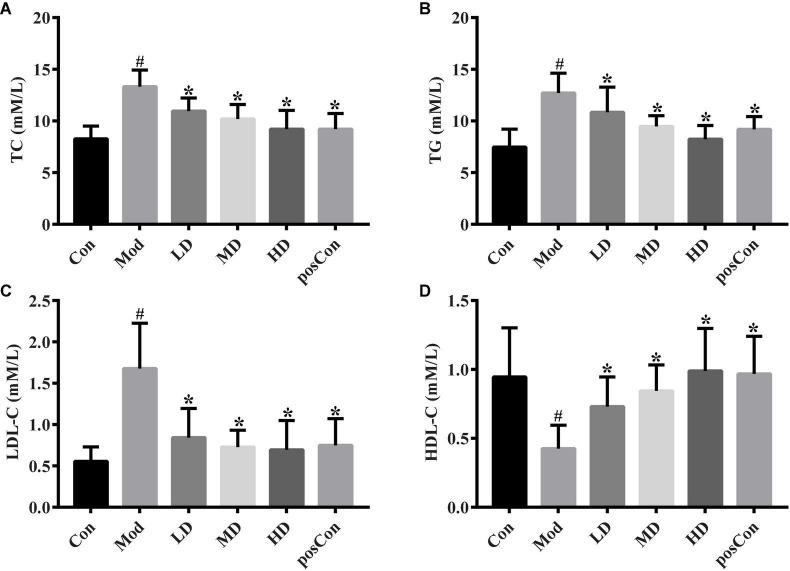
Altered plasma lipids by ACNE. The above bar graphs showed the plasma level of TC **(A)**, TG **(B)**, LDL-C **(C)**, and HDL-C **(D)**, respectively. The *p*-value less than 0.05 was considered statistical significance. ^#^*p* < 0.05, compared to the Con group; *p < 0.05, compared to the Mod group.

Common parameters of blood lipid were analyzed. In the Mod group, high-fat feeding induced a higher level of TC, TG, and LDL-C, along with a lower level of HDL-C, compared to the Con group (*p* < 0.05, Figure 1). Groups fed with ACNE (LD, MD, and HD) showed that dose-dependent significantly decreased plasma TC and TG levels and dose-dependent significantly increased plasma HDL-C levels, when compared to the Mod group (Figures 1A,B,D). ACNE resulted in significantly reduced plasma LDL-C level, but without a notable dose-dependent trend, with a comparison of the Mod group (Figure 1C). As a positive control drug, atorvastatin significantly decreased the levels of TC, TG, and LDL-C and elevated the level of HDL-C, compared to the Mod group.

### Metabolomics data processing and multivariate analysis

To explore the effects of supplementation with ACNE on plasma metabolic profiling, UHPLC-QTOF-MS-based non-targeted metabolomics was performed in both negative and positive ionization modes. Considering notable alterations being detected in plasma metabolites profile, samples from LD and MD were not subjected to metabolomics analysis. In total, 5 QC, 6 Con, 8 Mod, 8 HD, and 8 posCon samples were analyzed. The total ion chromatograms of five QC samples overlapped well (Supplementary Figure 3), suggesting good reproducibility in LC-MS analysis. After the conversion of raw data format, data processing was completed by R package XCMS. The 2,801 and 2,530 features were obtained from negative and positive ionization mode data, respectively. For data cleaning, noisy and abnormal features were filtered out, and 2,780 and 2,494 features corresponding to negative and positive ionization mode data were kept.

**FIGURE 2:**
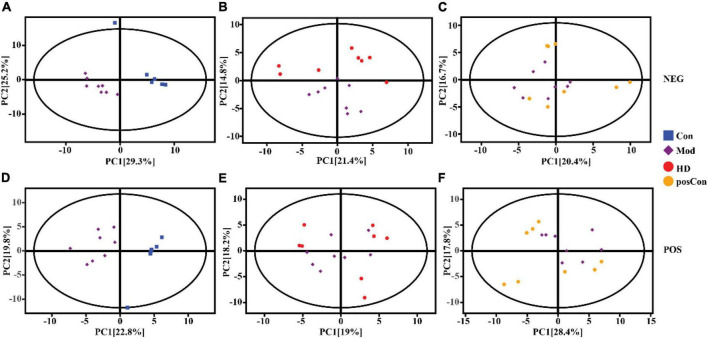
PCA score scatter plots showing clustering of plasma metabolites detected in ESI- mode (NEG) and ESI + mode (POS). **(A)** Mod vs. Con (NEG, R^2^X = 0.62), **(B)** HD vs. Mod (NEG, R^2^X = 0.58), **(C)** posCon vs. Mod (NEG, R^2^X = 0.51), **(D)** Mod vs. Con (POS, R^2^X = 0.55), **(E)** HD vs. Mod (POS, R^2^X = 0.59), **(F)** posCon vs. Mod (POS, R^2^X = 0.55).

**FIGURE 3:**
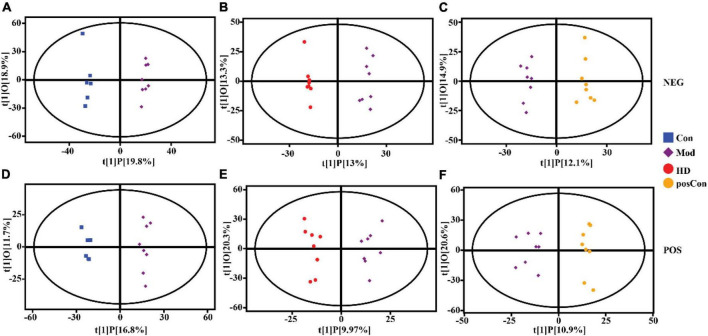
OPLS-DA score scatter plots showing clustering of plasma metabolites detected in ESI- mode (NEG) and ESI + mode (POS). **(A)** Mod vs. Con (NEG, R^2^Y = 0.99, Q^2^ = 0.87), **(B)** HD vs. Mod (NEG, R^2^Y = 0.99, Q^2^ = 0.77), **(C)** posCon vs. Mod (NEG, R^2^Y = 0.98, Q^2^ = 0.69), **(D)** Mod vs. Con (POS, R^2^Y = 0.99, Q^2^ = 0.78), **(E)** HD vs. Mod (POS, R^2^Y = 0.96, Q^2^ = 0.51), **(F)** posCon vs. Mod (POS, R^2^Y = 0.94, Q^2^ = 0.54).

Multivariate analysis including PCA and OPLS-DA was conducted separately on data acquired in both ionization modes with the SIMCA software (V16.0.2, Sartorius Stedim Data Analytics AB, Umea, Sweden). Based on the features determined in the negative ionization mode, a clear segregation between the Mod and Con groups was observed in the PCA score plot, as same as the HD and Mod groups, as shown in Figures 2A,B, while the posCon and Mod groups overlapped with each other (Figure 2C). For the positive ion mode data, the HD and Mod groups were interlaced, as well as samples between the posCon and Mod groups, as depicted in the PCA score plot (Figures 2E,F). Similar to negative ionization mode data, two clusters represented by the Mod and Con groups were separated by the PC1 component (Figure 2D). To exclude orthogonal variables unrelated to categorical/classifier variables, OPLS-DA was applied to screen the potentially important variables that contributed to the differentiation between groups. All OPLS-DA models were robust and high predictive, with minimum values of 0.94 for R2Y (cum) and 0.51 for Q2 (cum). Compared to PCA score plots, the segregation between the Mod group and other groups was enhanced for both ionization modes data along the vertical line representing the PC1 axis (Figure 3). The above results have shown substantial differences existing between the Mod group and other groups.

### Screening and identifying differential metabolites

**FIGURE 4:**
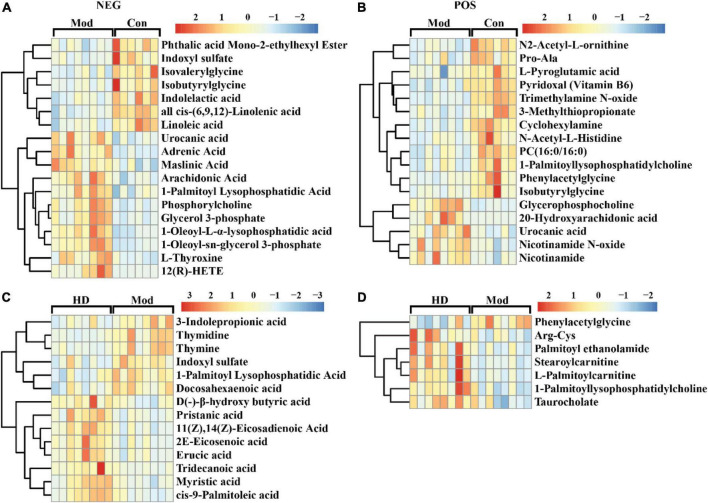
Heat map of the hierarchical clustering analysis of differential metabolites determined in ESI- mode (NEG) and ESI + mode (POS). **(A)** Mod vs. Con (NEG), **(B)** Mod vs. Con (POS), **(C)** HD vs. Mod (NEG), **(D)** HD vs. Mod (POS).

To recognize which variable/feature was mainly responsible for variation between the two groups, differential variables were initially screened according to variable importance to projections (VIP) value and the *p*-value generated from univariate analysis (Student’s *t*-test, VIP > 1, *p* < 0.05). The number of differential variables in both ionization modes (NEG/POS) between the Mod group and other groups was 612/470 (Con-Mod), 487/223 (HD-Mod), and 390/245 (posCon-Mod). The high-fat diet resulted in the maximum amounts of differential metabolites compared to the normal diet (Con group). In addition, the number of differential variables in negative ionization mode (NEG) is larger than that in POS possibly due to endogenous plasma metabolites being more suitable for detection in NEG mode. For identification of differential variables, retention time and mass to charge ratio (m/z) were searched against the local library (Biotree Ltd., Shanghai, China). A total of 416 and 668 variables hit the library with the input of 2,780 and 2,494 features corresponding to the NEG and POS modes. The identified differential metabolites were yielded by an intersection between differential variables and library-matched features. To screen more reliably identified differential metabolites, MS2 score > 0.75, VIP > 1.5, and FC (fold change) > 1.5 were further used for filtering library-mapped differential metabolites. Moreover, xenobiotic compounds such as drugs and chemical contaminants/pollutants were excluded by manual examination. Consequently, the final identified differential metabolites were listed in Supplementary Tables 1–3. The profile and level of differential metabolites were shown in the hierarchical cluster heatmap (Figure 4 and Supplementary Figure 4). As shown in Figures 4A,B, both urocanic acid and isobutyrylglycine were determined as differential metabolites between the Mod and Con groups in both ionization modes. Nevertheless, urocanic acid was significantly elevated in the Mod group and isobutyrylglycine was significantly decreased in the Mod group compared to the Con group. Furthermore, most differential metabolites between the Mod and Con groups were involved in lipid metabolism. It suggested that high-fat diet led to significant changes in lipid metabolism compared to the normal diet. Supplementation with a high dose of ACNE also resulted in significant changes in several metabolites associated with lipid metabolism, such as docosahexaenoic acid, palmitoyl ethanolamide, stearoylcarnitine, and L-palmitoylcarnitine (Figures 4C,D). Among these, three metabolites (indoxyl sulfate, 1-palmitoyl lysophosphatidylcholine, and phenylacetylglycine) were significantly affected by intervention with both high-fat diet and ACNE, as seen in Supplementary Figure 4D. Also, the alterations in the abundance of these differential metabolites were reflected by the peak integration area (Supplementary Figure 5). For comparison of the HD and posCon groups, there were only a few differential metabolites including sucrose, N-acetyl-L-tyrosine, D-quinovose, indoxyl sulfate, and mevalonic acid 5-phosphate (Supplementary Figures 4C, 5I).

#### Metabolic pathway analysis

**FIGURE 5:**
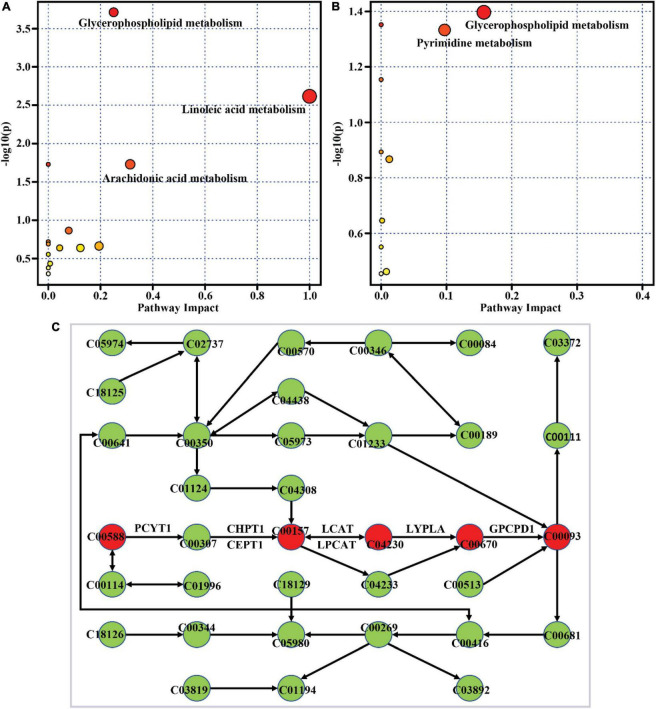
Metabolic pathway enrichment of differential metabolites. **(A)** Mod vs. Con, **(B)** HD vs. Mod, **(C)** the enriched glycerophospholipid metabolism pathway generated by MetaboAnalyst. The red circle indicates the enriched differential metabolite. C00588, Choline phosphate; C00307, CDP-choline; C00157, Phosphatidylcholine; C04230, 1-Acyl-sn-glycero-3-phosphocholine; C00670, Glycerophosphocholine; C00093, Glycerophosphoric acid; PCYT1, choline phosphate cytidylyltransferase; CHPT1, diacylglycerol choline phosphotransferase; CEPT1, choline/ethanolamine phosphotransferase; LCAT, lecithin-cholesterol acyltransferase; LPCAT, lysophosphatidylcholine acyltransferase/lyso-PAF acetyltransferase; LYPLA1, lysophospholipase I; GPCPD1, glycerophosphocholine phosphodiesterase.

To clarify the metabolic pathway of differential metabolites, metabolic pathway enrichment analysis was performed with the MetaboAnalyst tool. The result showed glycerophospholipid metabolism, linoleic acid metabolism, and arachidonic acid metabolism as the most significantly altered pathways with high-impact values when comparing the Mod group to the Con group, as depicted in Figure 5A. The differential metabolites between the HD group and the Mod group were significantly enriched in glycerophospholipid metabolism and pyrimidine metabolism (Figure 5B). Both high-fat diet and ACNE supplementation had significant impacts on glycerophospholipid metabolism. The overview of this pathway with enriched differential metabolites and their related metabolic enzymes was illustrated in Figure 5C. It was shown that choline-related metabolites were significantly enriched in the glycerophospholipid metabolism pathway.

### Analysis of interactions between proteins related to metabolites

**FIGURE 6:**
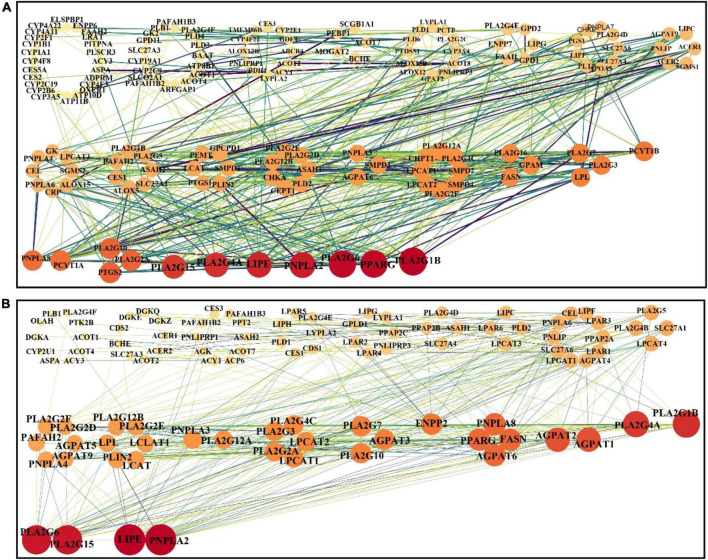
PPIs network analysis of differential metabolites related proteins. **(A)** Mod vs. Con, **(B)** HD vs. Mod.

To identify the possible interactions between metabolite and its related proteins, the association of metabolite and protein and the relations between proteins were analyzed according to the previously described approach (21). Owing to most differential metabolites being lipid-related metabolites, the associated proteins of the predominant lipid-related differential metabolites (listed in Supplementary Table 4) were extracted from HMDB (version 5.0) via in-house R script. There were 206 and 129 proteins associated with lipid-related differential metabolites between the Mod and Con groups and between the HD and Mod groups, respectively (seen in Supplementary Figures 6A,B). Five shared lipid-related differential metabolites between the HD-Mod and posCon-Mod (highlighted in red color in Supplementary Table 4) were found and 52 related proteins were extracted, as illustrated in Supplementary Figure 6C. The protein-protein interaction (PPI) network was built by the online tool STRING (v11.0) and analyzed by Cytoscape 3.8. The results were visualized in Figures 6A,B, and Supplementary Figure 6D. The identified hub proteins included PLA2G1B (P04054), PPARG (P37231), PLA2G6 (O60733), PNPLA2 (Q96AD5), LIPE (Q05469), PLA2G15 (Q8NCC3), AGPAT1 (Q99943), and ENPP2 (Q13822). PLA2G1B (group I phospholipase A2), PPARG (peroxisome proliferator-activated receptor gamma), and PLA2G6 (group VI phospholipase A2) were associated with linoleic acid (HMDB0000673), glycerol 3-phosphate (HMDB0000126), and arachidonic acid (HMDB0001043), respectively, which were significantly changed by a high-fat diet. Supplementation with ACNE had significant effects on the abundance of myristic acid (HMDB0000806) and stearoylcarnitine (HMDB0000848), which were linked with PNPLA2 (patatin-like phospholipase domain-containing protein 2) and PLA2G15 (group XV phospholipase A2), respectively. Also, myristic acid might associate with LIPE (hormone-sensitive lipase). Both interventions with ACNE and atorvastatin exerted a significant impact on 1-oleoyl-L-α-lysophosphatidic acid (HMDB0007855), which were associated with AGPAT1 (1-acyl-sn-glycerol-3-phosphate acyltransferase alpha) and ENPP2 (ectonucleotide pyrophosphatase/phosphodiesterase family member 2).

## Discussion

Anthocyanins are ubiquitously present in fruits, vegetables, and related processed products such as jams, juices, and beverages, and are responsible for various colors, tastes, and flavors, along with a variety of promoting health effects. The available health benefits of anthocyanins, for instance, protective effects against diabetes mellitus (17) and cardiovascular diseases (14), mainly arise from antioxidant and anti-inflammatory properties. Existing experimental evidence supports that anthocyanins and their metabolites are favorable to alleviating atherosclerosis (22), which is characterized by the occurrence of lipid deposition in medium-large arterial walls mainly resulting from dysfunction in lipid metabolism and chronic inflammation. Recently, studies on anthocyanins of cherries and their related products have been increasing for exploring the color formation and health benefits via multiple omics technologies such as metabolomics and transcriptomics (16). Nevertheless, results from some anthocyanins-dietary intervention experiments on animal models and human subjects are not consistent. It might be due to the different dose and duration of intervention, as well as the bioavailability and purity of anthocyanins. Moreover, the molecular mechanism behind the beneficial effects of anthocyanins is still not fully clarified. In the present study, the mechanism underlying protective effects against atherosclerosis of anthocyanins derived from Xinjiang wild cherry plum peel was preliminarily explored by global plasma metabolomics based on LC-MS.

ApoE-deficient mouse is a commonly used animal model for atherosclerosis, which is predisposed by obesity and inflammation. Although previous studies have shown that anthocyanins could reduce the body weight of high-fat-fed mice (17), body weight loss has not been dramatically obtained after 18 weeks of intervention with ACNE in our experimental results, as shown in Supplementary Figure 1B. This might be suggested by similar body weight gain on 12-week high-fat diet compared to a normal diet in apoE^–/–^ mouse, which is possibly explained by differences in feeding diet and mouse strain because non-genetic modified mice (wild-type) were used in most previous studies on anthocyanins. Despite no significant difference in body weight between the Mod and Con groups, the establishment of the atherosclerosis model was confirmed by other metrics or methods, such as inflammatory factors, regular biochemical analysis of plasma lipids, and histological assessment of aortic arch (data not shown). As mentioned in the above results, a high-fat diet resulted in the increased circulating level of inflammatory marker (C-reactive protein, CRP, Supplementary Figure 1C), lipid peroxidation marker (malondialdehyde, MDA, Supplementary Figure 2D), blood lipids (TC, TG, LDL-C, Figures 1A–C), liver enzymes (ALT, AST, Supplementary Figure 7), and decreased circulating level of HDL-C (Figure 1D), as well as antioxidant activity (SOD, TAOC, GPx, Supplementary Figures 2A–C), which at least partially demonstrated the successful development of an animal model for atherosclerosis. After supplementation with ACNE for 18 weeks, changes in these metrics were reversed. These findings were consistent with previously reported results (23).

Global LC-MS metabolomic analysis of plasma from apoE^–/–^ mice indicated that ACNE affected metabolite profile. Several significantly differential metabolites were identified. Compared to the normal diet, a high-fat diet significantly altered the level of urocanic acid and isobutyrylglycine, which were both determined in negative and positive ionization modes (Figures 4A,B). Urocanic acid is a degradation product of histidine, possessing multiple roles in health and disease (24). It has two isomers, cis- and trans-form, presented in skin, brain, urine, feces, and plasma, and is commonly identified as differential metabolites, implying its potential implication in various physiological and pathological processes. Isobutyrylglycine, a member of short-chain acylglycine, is produced in the catabolism of leucine, isoleucine, and valine. In a recent study of urine metabolome of adolescent boys and girls, isobutyrylglycine was found to be related to body composition (BMI and body fat) in males, exhibiting decreased levels with higher body composition (25). Similarly, the alteration in the level of isovalerylglycine was the same as that of isobutyrylglycine. In our current study, a high-fat diet also significantly reduced the level of isovalerylglycine (Figure 4A) compared to the normal diet. These findings enhance our confidence in the reliability of the identification of differential metabolites. Among the differential metabolites between the Mod and Con groups, most were associated with lipid metabolism, suggesting an evidently reasonable link with the high-fat diet.

In the comparison of the HD group with the Mod group, there were several differential metabolites influenced by ACNE, along with the majority of lipid metabolism-related metabolites. Among these, indoxyl sulfate,1-palmitoyllysophosphatidylcholine, and phenylacetylglycine were the shared differential metabolites between Mod-Con and HD-Mod. Indoxyl sulfate (IS) is produced from tryptophan metabolism via the indole metabolic pathway, with the predominant contribution of gut microbiota, including *E. coli* (26). IS, as one of the main uremic toxins, is extensively studied in the aspect of the pathogenesis of cardiovascular diseases during chronic kidney disease (CKD). IS, also known as endotheliotoxin, exerts important actions in the dysfunction of endothelium, which is usually the first event of atherosclerosis and thrombosis (27). Recently, a study has demonstrated that IS induces proinflammatory activation of macrophages and accelerates atherogenesis in CKD (28). The 1-Palmitoyl-lysophosphatidylcholine (LPC16:0) belongs to lysophosphatidylcholines (LPCs) known as proinflammatory lipid mediators. LPC16:0 could stimulate the formation of proinflammatory mediators encompassing IL-5, IL-6, NO, 12-HETE, and PGE2 (29). N-phenylacetylglycine (PAGly) is a host-microbial co-metabolite, with production from glycine conjugation to phenylacetic acid which is converted from phenylalanine in the large intestine of rodents by gut microbes (30). However, in recent studies, PAGly was also determined as a differential metabolite in a human urine sample (31). Initially, PAGly was identified as a biomarker of drug-induced phospholipidosis, whereby the accumulation of excess phospholipids occurs within the cell, especially in the lysosome (30). Recently, PAGly and its equivalent in humans (N-phenylacetyl-glutamine, PAGln) were found to promote cardiovascular disease via adrenergic receptors (32). Interestingly, PAGly has also been reported to exhibit protective effects against cardiac injury resulting from ischemia/reperfusion through activating β2AR (33). Both IS and PAGly are influenced by gut microbes, along with similar pattern of alteration in abundances (reflected by peak area), as seen in Supplementary Figure 5. These findings prompt that high-fat diet and ACNE possibly have impact on the interaction between host and gut microbes.

According to the results of metabolic pathway analysis, a high-fat diet and ACNE primarily influenced glycerophospholipid metabolism and linoleic acid metabolism. In the pathway of glycerophospholipid metabolism (Figure 5C), ACNE might alter the activities or expression of some metabolic enzymes, such as lecithin-cholesterol acyltransferase (LCAT), lysophosphatidylcholine acyltransferase/lyso-PAF acetyl-transferase (LPCAT), lysophospholipase I (LYPLA1), and glycerophosphocholine phosphodiesterase (GPCPD1), which further change the level of relevant metabolites. To our knowledge, it has not shown the effect of anthocyanins on these enzymes. Thus, it is necessary to conduct further study on this aspect. From a perspective of the relationship between metabolite and protein, several important proteins (enzymes) were identified for partially explaining the anti-atherogenicity of ACNE. Previously, a study indicated that anthocyanins (cyanidin 3-glucoside; C3G) could upregulate the gene expression of LIPE (also known as hormone-sensitive lipase, HSL) and enhance the lipolytic activity in adipocytes isolated from rat (34). Nevertheless, several studies have shown inhibitory effects of anthocyanins on lipolytic activity, along with downregulation of PNPLA2 (also known as adipose triglyceride lipase, ATGL) and PPARG (peroxisome proliferators-activated receptor-γ) under conditions of hyperglycemia and differentiation in preadipocyte 3T3-L1 (35). The contradictory results might imply the effect of anthocyanins varies with the cell under different conditions. Additionally, anthocyanidins (cyanidin, malvidin, peonidin, petunidin, and delphinidin) have been reported to inhibit the activity of PLA2 (phospholipase A2) (36). As seen in Supplementary Figure 6D, ENPP2 and AGPAT1 may implicate in the alteration of lipid metabolism by both ACNE and atorvastatin. ENPP2 is an adipose-derived, secreted enzyme that catalyzes lysophosphatidylcholine to form lysophosphatidic acid, a biological molecule possessing various biological effects. ENPP2 has been reported implication in multiple physiopathological processes, such as dendritic cell migration (37), adipose tissue expansion, and insulin resistance (38). AGPAT1, as an intermediate enzyme in the pathway for the biosynthesis of glycerophospholipids, catalyzes the acylation of lysophosphatidic acid to produce phosphatidic acid (39). Thereby, based on the above analyses, we propose an inference that the obtained benefits of ACNE on apoE-deficient mice fed with a high-fat diet predominantly result from alterations in the expression or activity of phospholipids-related proteins, such as LCAT, LPCAT, LYPLA1, GPCPD1, PLA2G1B, PPARG, PLA2G6, LIPE, PNPLA2, AGPAT1, and ENPP2. So, there are other experiments required for further confirmation.

Our work is a preliminary study regarding the effects of ACNE on the early stage of atherosclerosis. There are some limitations to this study. In one hand, the identification of differential metabolite was not validated by spiking authenticated standards. On the other hand, the possible mechanisms were not further confirmed by molecular biology techniques. Furthermore, despite the composition of ACNE was characterized by our previous study, the plasma metabolites of anthocyanins were not analyzed in the present study. Next, the future research direction will be focused on the following aspects: (i) confirmation of the identity of key differential metabolites with authenticated standards; (ii) exploring the influence of ACNE on the expression and activity of key proteins and identifying the true target protein of anthocyanins with *in silico* and experimental approaches; and (iii) clarifying the role of host-microbial co-metabolites affected by anthocyanins in the early stage of atherosclerosis development.

In conclusion, a high-fat diet can lead to marked alterations in a series of plasma metabolites mainly related to lipid metabolism in apoE-deficient mice. These differential metabolites are primarily enriched in the pathway of glycerophospholipid metabolism and linoleic acid metabolism. Feeding with ACNE from Xinjiang wild cherry plum peel can ameliorate or reverse many of these changes. Metabolic pathway analysis and construction of PPI network based on differential metabolites-related proteins have uncovered a panel of key proteins possibly implicated in the protective effects of ACNE against atherosclerosis. Moreover, the identification of some host-microbial co-metabolites has suggested that ACNE can modulate the intestinal flora, altering the level of metabolites involved in atherosclerosis. To the best of our knowledge, it is the first time to investigate the anti-atherogenic effect of anthocyanins from Xinjiang wild cherry plum peel by metabolomics technique. This extends our understanding of the molecular mechanisms behind the protective effects of anthocyanins against atherosclerosis and offers new insights into the exploitation of Xinjiang wild cherry plum.

## Data availability statement

The original contributions presented in this study are included in the article/Supplementary material, further inquiries can be directed to the corresponding author/s.

## Ethics statement

The animal study was reviewed and approved by Committee on Animal Research and Ethics of Xinjiang Medical University.

## Author contributions

JS: conceptualization, methodology, validation, investigation, formal analysis, and writing–original draft. XL: data processing and analysis, visualization, and writing–original draft. XZ: methodology and writing–review and editing. ZL, GA, and YL: methodology, validation, and writing–review and editing. JY: methodology, writing–review and editing, supervision, project administration, and funding acquisition. PZ: methodology, writing–review and editing, and supervision. All authors contributed to the article and approved the submitted version.

## Footnotes

1
www.hmdb.ca

2
www.genome.jp/kegg/

3
https://pubchem.ncbi.nlm.nih.gov/

4
http://metlin.scripps.edu
