# Supplementary material for: Effects of Xinjiang wild cherry plum (Prunus divaricata Ledeb) anthocyanin-rich extract on the plasma metabolome of atherosclerotic apoE-deficient mice fed a high-fat diet
Source: Front Nutr. 2022 Jul 25;9:923699. doi: 10.3389/fnut.2022.923699 (PMC9358619; doi:10.3389/fnut.2022.923699)
Supplement: Supplementary file 1 [file Data_Sheet_1.pdf]

Supplementary material

Supplementary Methods

Preparation of anthocyanin extract (ACNE) and compositional analysis. The fresh fruit peel of wild cherry plum was collected from Daxigou town belonging to Huocheng County, Ili, Xinjiang, China, and flash froze, stored. Anthocyanin extract was prepared according to the approaches described in our previous study published in Chinese[13]. Briefly, the frozen plum peel was ground and transferred into a glass beaker. Then, an acidified solution of methanol (1% HCl) was added to the beaker according a solid-liquid ratio of 1:5 (w/v) and the beaker was placed into the hot water bath kept 55℃ for 80 min. After that, the solution was filtered through and the filtrate was collected. For the second round of extraction, a half volume of extracting solvent was added to the remnant and extraction was done with the same condition as the first round of extraction. The two filtrates were combined, centrifuged and the supernatant was collected, concentrated to remove methanol using a vacuum-concentrated rotary evaporator at 40 °C. After evaporation, the concentrate was loaded onto a column packed with an equilibrated HP2MGL adsorbent (DIAIONTM, Mitsubishi Chemical Corporation, Japan) for purification. To remove proteins, polysaccharides and other water-soluble impurities, the column was first eluted with distilled water until the eluent was clear. Subsequently, anthocyanins were eluted with acidified ethanol-water solution (80% v/v, pH 2.5) at a flow velocity of 2.1 ml/min, and the collected eluent was subjected to vacuum rotary evaporation to remove ethanol. The total anthocyanin content of purified extract was determined by a pH-differential method[29].

The compositional analysis of anthocyanin extract was performed by high-resolution LC-ESI-Orbitrap-MS. The extract was separated by an Agilent 1220 HPLC system (Agilent Technologies, Palo Alto, CA) equipped with a reversed-phase C18 column (250 mm×3.0 mm, 5 μm; Waters). The mobile phase was composed of 100% acetonitrile and water containing 2% formic acid. The separation procedure was run under the following conditions: column temperature, 35℃; flow rate, 0.3 ml/min; injection volume, 20 μl. The HPLC system was hyphenated with Orbitrap Fusion Lumos Mass Spectrometer (Thermo Fisher Scientific, San Jose, CA, USA) equipped with an electrospray ion source (ESI). Full-scan MS spectra were acquired over a range of m/z 100-1100 in positive mode. The operation conditions were listed as follows: nebulizing gas (N_2), 345 kPa; drying gas (N_2), 300℃, 30 L/min; capillary voltage, 3000 V; fragmentation voltage, 40 eV. Anthocyanins were tentatively identified by accurate mass of molecular ion and comparison of existing data from published papers, and confirmed by tandem MS (MS/MS) data.

Supplementary Figures

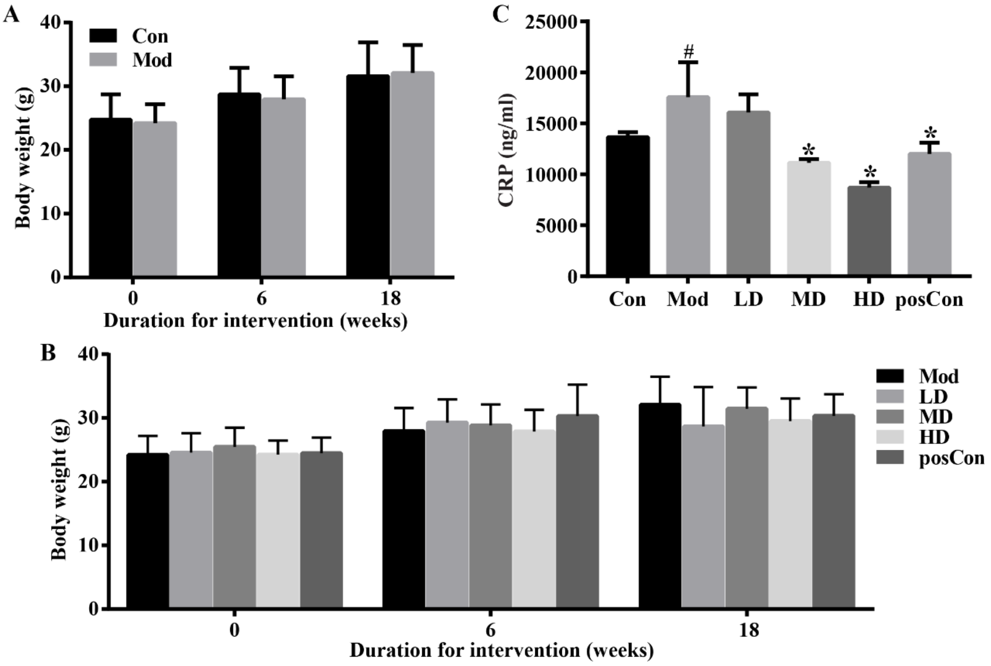

Figure 1 The influence of ACNE on body weight and plasma CRP. Changes in body weights between the Con and Mod groups (A), between the treatment groups (LD, MD, HD, posCon) and Mod groups (B) were showed by bar graphs. Changes in the plasma level of CRP among groups were showed in bar graph (C). The p value less than 0.05 was considered as statistical significance. #: p<0.05, comparing to the Con group; *: p<0.05, comparing to the Mod group.

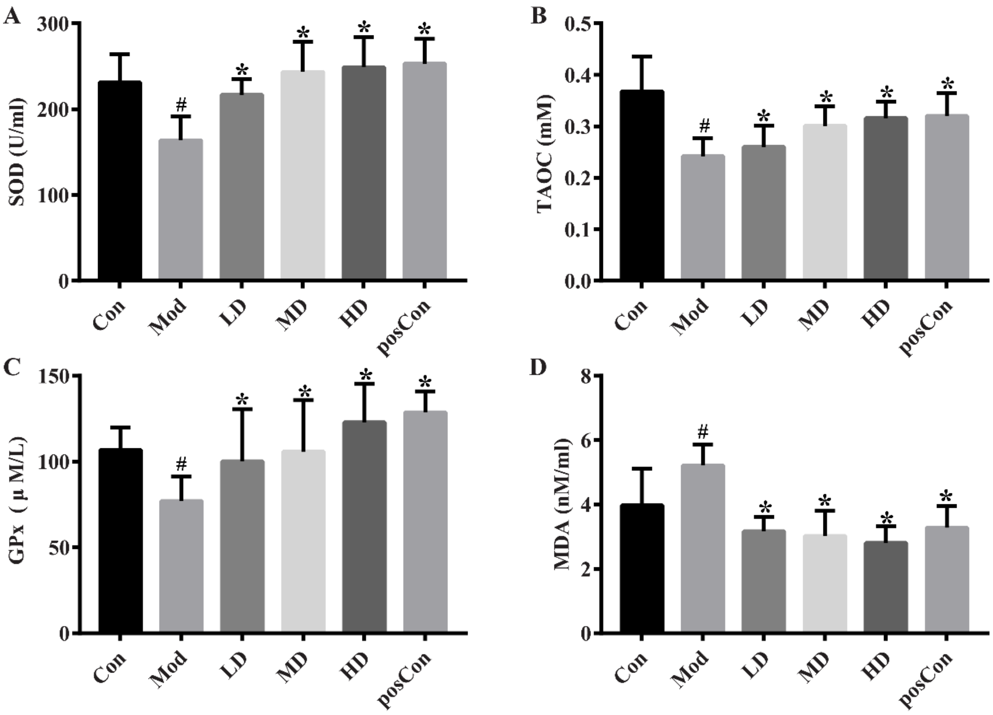

Figure 2 Altered antioxidant capacity in plasma by ACNE. The above bar graphs showed the plasma level of SOD (A), TAOC (B), GPx (C), and MDA (D), respectively. The p value less than 0.05 was

considered as statistical significance. #: p<0.05, comparing to the Con group; *: p<0.05, comparing to the Mod group.

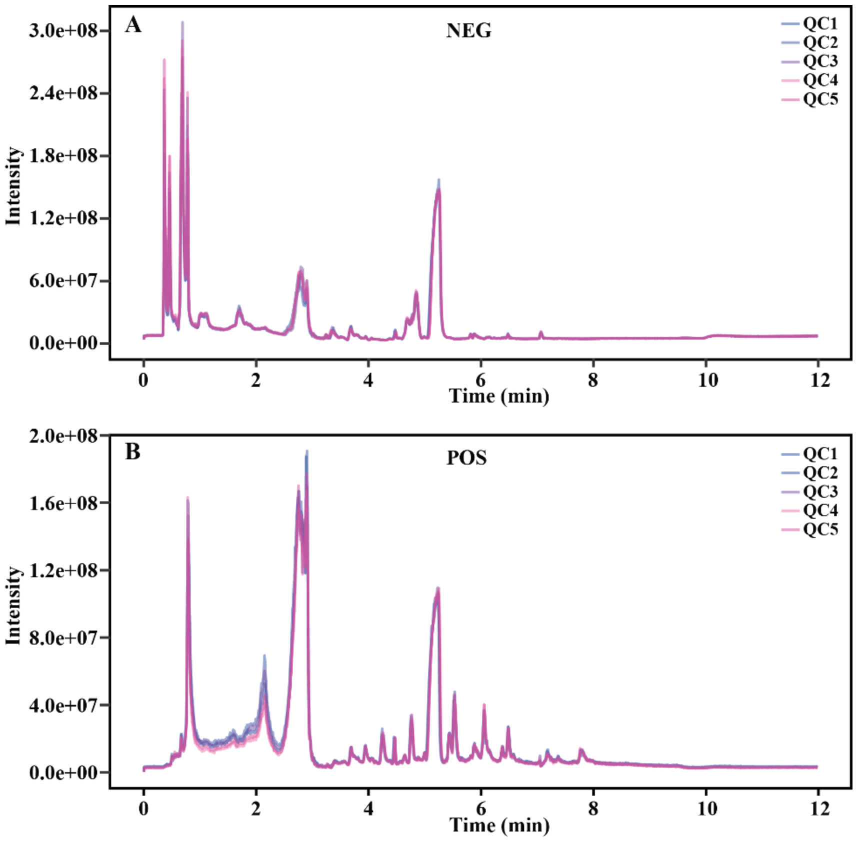

Figure 3 Total ion chromatogram of QC samples. (A) in negative ionization mode (NEG), (B) in positive ionization mode (POS).

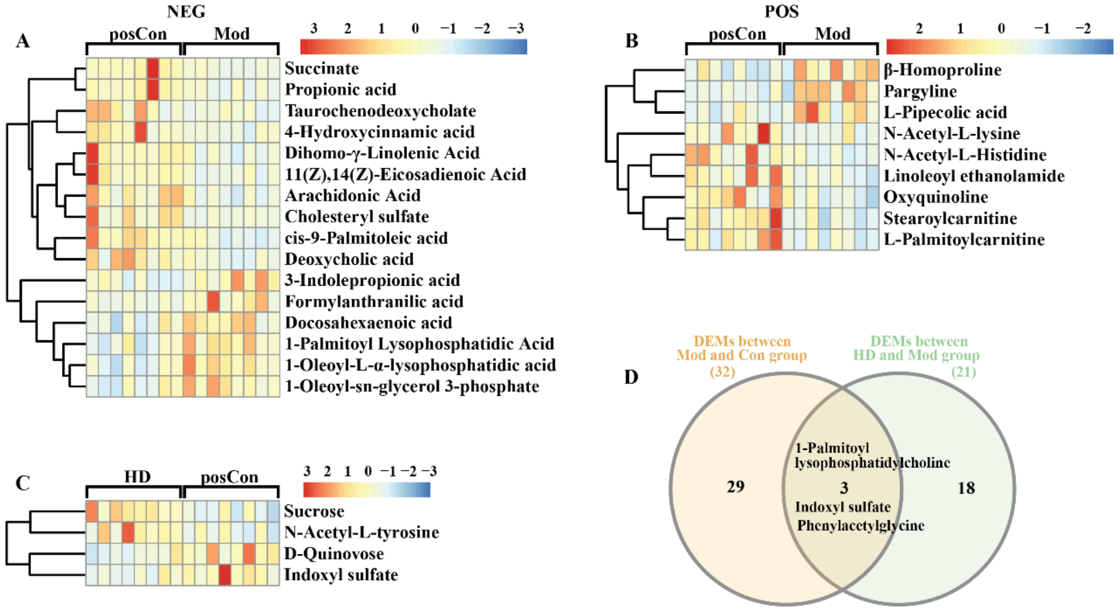

Figure 4 Heat map of the hierarchical clustering analysis of differential metabolites determined in ESI- mode (NEG) and ESI+ mode (POS). (A) posCon vs Mod (NEG), (B) posCon vs Mod (POS),

(C) HD vs posCon (NEG), (D) the Venn plot depicting the shared differential metabolites. The Venn plot was plotted by using web-based program, InteractiVenn (http://www.interactivenn.net/).

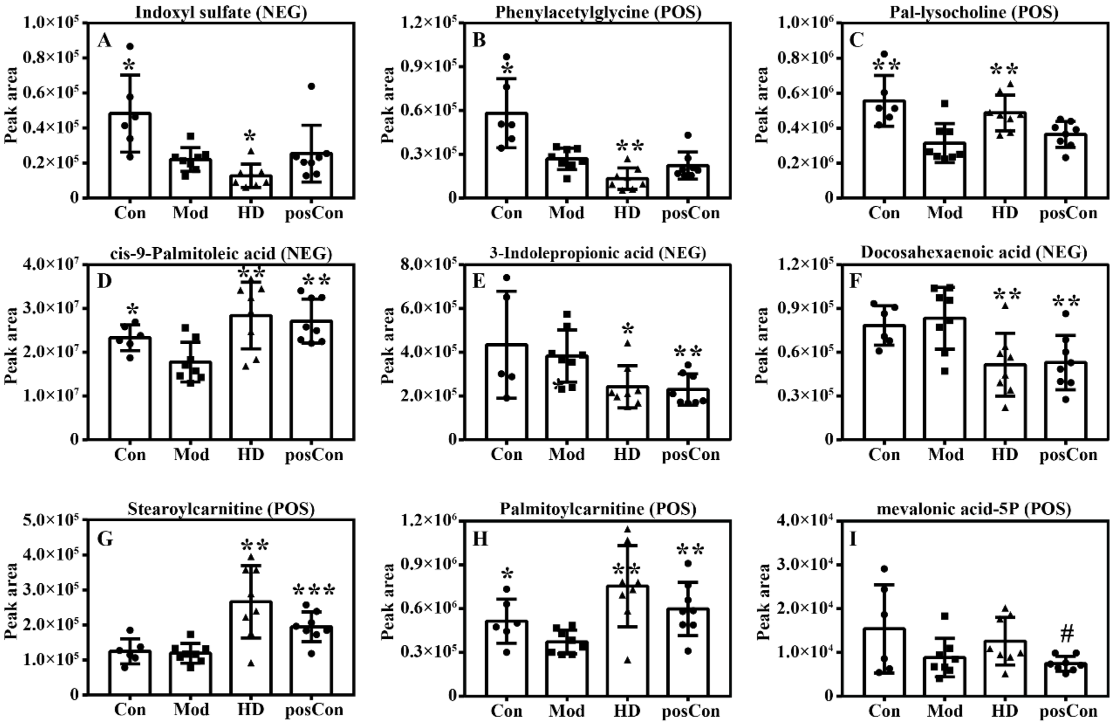

Figure 5 The peak areas of some selected differential metabolites under the curve of extracted ion chromatogram. (A) Indoxyl sulfate, (B) Phenylacetylglycine, (C) 1-Palmitoyl lysophosphatidic acid, (D) cis-9-Palmitoleic acid, (E) 3-Indolepropionic acid, (F) Docosahexaenoic acid, (G) Stearoylcarnitine, (H)Palmitoylcarnitine, (I) Mevalonic acid-5 phosphate.

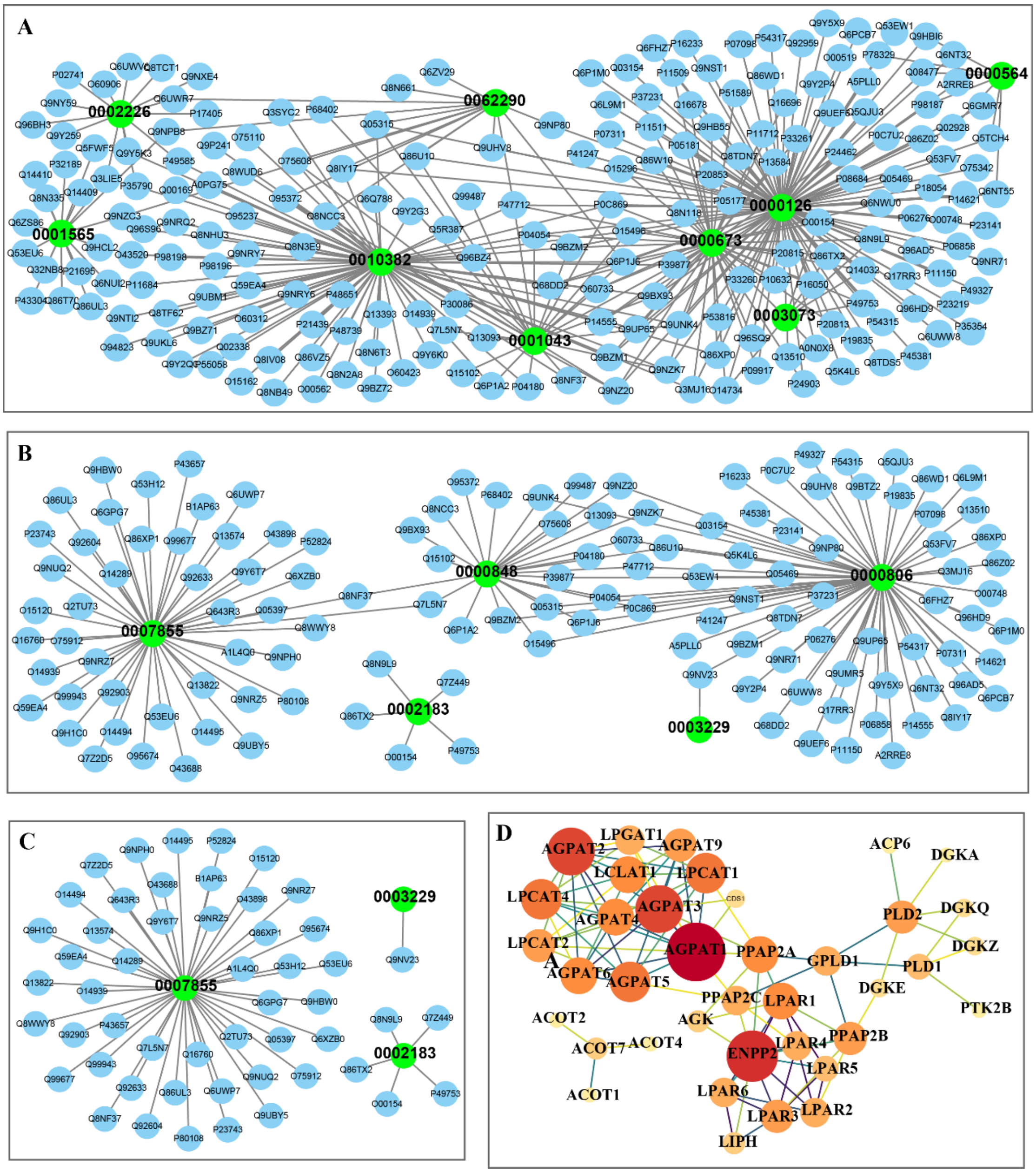

Figure 6 The proteins associated with the main differential lipid metabolites and PPIs network analysis. (A) The primary lipid differential metabolites between the Mod and Con group as well as their related proteins. (B) The primary lipid differential metabolites between the HD and Mod group as well as their related proteins. (C) The shared lipid differential metabolites between HD-Mod and posCon-Mod as well as their related proteins. (D) PPIs network between proteins associated with the common lipid differential metabolites between HD-Mod and posCon-Mod. The green circle indicates the accession number (hmdb_ID) of metabolites in HMDB, as listed in **Supplementary Tables**.

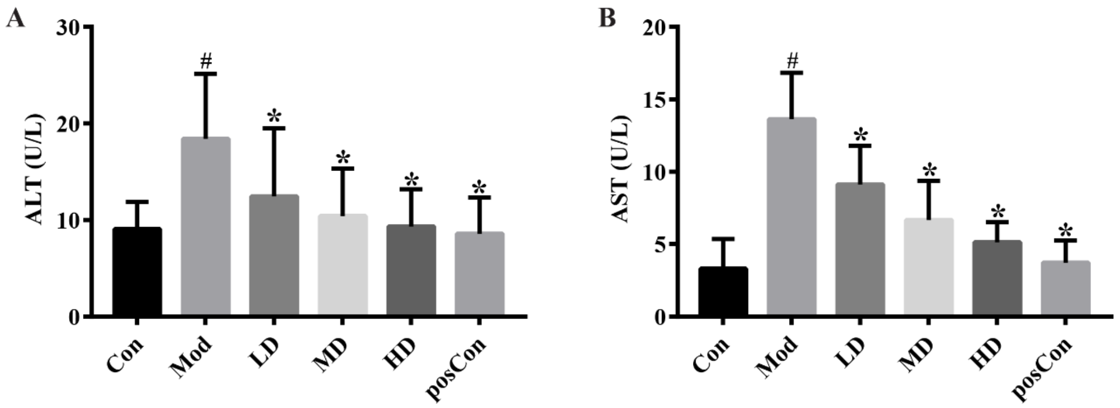

Figure 7 The effects of ACNE on ALT and AST. The above bar graphs showed the level of ALT (A) and AST (B), respectively. The p value less than 0.05 was considered as statistical significance. #: p<0.05, comparing to the Con group; *: p<0.05, comparing to the Mod group.

Supplementary Tables

Table S1 Differential metabolites between the Mod group and the Con group.

No.	Metabolite	P-Value	VIP	FC	MS2.score	hmdbID
	**Negative ion mode**					
1	all cis-(6,9,12)-Linolenic acid	1.41E-06	1.97	0.54	0.9938	HMDB0003073
2	1-Oleoyl-L-α-lysophosphatidic acid	1.92E-04	1.99	3.27	0.9903	--
3	Linoleic acid	3.44E-04	1.78	0.62	0.9998	HMDB0000673
4	1-Oleoyl-sn-glycerol 3-phosphate	5.57E-04	2.02	4.31	0.9821	--
5	7,10,13,16-Docosatetraenoic acid	6.42E-04	2.00	2.96	0.9984	HMDB0002226
6	Isovalerylglycine	7.64E-04	1.86	0.50	0.9956	HMDB0000678
7	Indolelactic acid	8.79E-04	1.78	0.66	0.96	HMDB0000671
8	Phthalic acid Mono-2-ethylhexyl Ester	1.07E-03	1.55	0.40	0.7867	HMDB0013248
9	Phosphorylcholine	2.03E-03	1.76	1.56	0.9757	HMDB0001565
10	Maslinic Acid	3.41E-03	1.68	1.55	0.8193	HMDB0002392
11	L-Thyroxine	3.44E-03	1.63	2.10	0.9327	HMDB0000248
12	Glycerol 3-phosphate	3.52E-03	1.69	1.52	1	HMDB0000126
13	Urocanic acid	4.51E-03	1.65	1.59	0.9441	HMDB0000301
14	Arachidonic Acid	5.12E-03	1.63	1.79	0.9741	HMDB0001043
15	1-Palmitoyl Lysophosphatidic Acid	7.29E-03	1.56	1.66	0.9955	HMDB0010382
16	12R-HETE	1.86E-02	1.75	8.85	0.9281	HMDB0062290
17	Indoxyl sulfate	2.28E-02	1.69	0.46	0.9133	HMDB0000682
18	Isobutyrylglycine	2.87E-02	1.83	0.48	0.9943	HMDB0000730
	**Positive ion mode**					
1	Trimethylamine N-oxide	3.21E-08	2.29	0.30	0.9919	HMDB0000925
2	Cyclohexylamine	3.56E-05	2.18	0.57	0.7894	HMDB0031404

3	N2-Acetyl-L-ornithine	4.27E-05	2.14	0.56	0.9318	HMDB0003357
4	Pyridoxal (Vitamin B6)	1.88E-04	2.00	0.44	0.9273	HMDB0000239
5	PC (16:0/16:0)	3.35E-04	1.88	0.42	0.9978	HMDB0000564
6	3-Methylthiopropionate	7.84E-04	2.04	0.58	0.9293	HMDB0001527
7	Glycerophosphocholine	1.43E-03	2.05	1.84	0.9913	HMDB0000086
8	1-Palmitoyllysophosphatidylcholine	3.40E-03	1.81	0.54	0.7967	HMDB0010382
9	L-Pyroglutamic acid	3.88E-03	1.51	0.66	0.9951	HMDB0000267
10	Nicotinamide N-oxide	7.11E-03	1.67	1.96	0.9439	HMDB0002730
11	Nicotinamide	1.25E-02	1.62	1.67	0.9984	HMDB0001406
12	Pro-Ala	1.32E-02	1.52	0.63	0.92	HMDB0029010
13	Urocanic acid	1.43E-02	1.57	1.52	0.9873	HMDB0000301
14	Phenylacetylglycine	2.08E-02	1.93	0.43	0.9997	HMDB0000821
15	N-Acetyl-L-Histidine	2.41E-02	1.81	0.45	0.9958	HMDB0032055
16	20-Hydroxyarachidonic acid	2.63E-02	1.79	7.61	0.8566	HMDB0005998
17	Isobutyrylglycine	3.49E-02	1.87	0.61	0.9902	HMDB0000730

Table S2 Differential metabolites between the HD group and the Mod group.

No.	Metabolite	P-Value	VIP	FC	MS2.score	hmdbID
	**Negative ion mode**					
1	D(-)-beta-hydroxy butyric acid	2.80E-02	1.51	1.74	0.8727	HMDB0000011
2	Thymine	6.22E-03	2.06	0.67	0.9993	HMDB0000262
3	3-Indolepropionic acid	2.86E-02	1.67	0.64	0.9731	HMDB0002302
4	Indoxyl sulfate	1.34E-02	1.78	0.59	0.9133	HMDB0000682
5	Tridecanoic acid (Tridecylic acid)	3.98E-02	1.79	1.73	0.9439	HMDB0000910
6	Myristic acid	3.71E-03	1.77	1.53	0.9997	HMDB0000806
7	Thymidine	2.80E-03	2.27	0.55	0.8021	HMDB0000273
8	cis-9-Palmitoleic acid	4.48E-03	1.76	1.61	1	HMDB0003229
9	Pristanic acid	4.63E-03	1.87	1.85	0.9558	HMDB0000795
10	11(Z),14(Z)-Eicosadienoic Acid	1.22E-03	2.01	1.61	0.9975	--
11	2E-Eicosenoic acid	5.80E-03	1.87	1.56	0.9999	--
12	Docosahexaenoic acid	1.35E-02	1.72	0.63	0.9984	HMDB0002183
13	Erucic acid	1.24E-02	1.71	1.61	0.9811	HMDB0002068
14	1-Palmitoyl Lysophosphatidic Acid	1.26E-02	1.78	0.61	0.9955	HMDB0007855
	**Positive ion mode**					
1	Phenylacetylglycine	6.22E-03	2.07	0.52	0.9997	HMDB0000821
2	Stearoylcarnitine	7.82E-03	2.02	2.37	0.9981	HMDB0000848
3	L-Palmitoylcarnitine	9.01E-03	1.93	2.15	0.9952	HMDB0240774
4	1-Palmitoyllysophosphatidylcholine	1.40E-02	2.02	1.62	0.7967	HMDB0010382
5	Taurocholate	1.89E-02	1.73	1.80	0.9097	HMDB0000036
6	Palmitoyl ethanolamide	2.67E-02	1.56	1.92	0.9874	HMDB0002100
7	Arg-Cys	4.55E-02	1.61	1.65	0.8773	HMDB0028706

Table S3 Differential metabolites between the posCon group and the Mod group.

No.	Metabolite	P-Value	VIP	FC	MS2.score	hmdbID
	**Negative ion mode**					
1	cis-9-Palmitoleic acid	0.003132	2.03	1.57	1	HMDB0003229
2	1-Oleoyl-L-α-lysophosphatidic acid	0.003939	2.06	0.55	0.9903	HMDB0007855
3	1-Palmitoyl Lysophosphatidic Acid	0.009456	1.81	0.66	0.9955	HMDB0007853
4	3-Indolepropionic acid	0.010171	1.87	0.61	0.9731	HMDB0002302
5	Arachidonic Acid	0.010439	1.62	1.64	0.9741	HMDB0001043
6	Docosahexaenoic acid	0.011368	1.90	0.65	0.9984	HMDB0002183
7	Deoxycholic acid	0.018828	1.81	1.87	0.9997	HMDB0000626
8	1-Oleoyl-sn-glycerol 3-phosphate	0.020145	1.76	0.55	0.9821	--
9	Taurochenodeoxycholate	0.022468	1.60	2.45	1	HMDB0000951
10	Dihomo-γ-Linolenic Acid	0.025806	1.79	1.77	0.996	HMDB0002925
11	Cholesteryl sulfate	0.026295	1.79	1.80	0.9975	HMDB0000653
12	Succinate	0.027292	2.17	2.03	0.9638	HMDB0000254
13	11(Z),14(Z)-Eicosadienoic Acid	0.028639	1.76	1.63	0.9975	HMDB0005060
14	Propionic acid	0.031343	1.89	1.53	0.998	HMDB0000237
15	4-Hydroxycinnamic acid	0.032938	1.75	1.58	0.9974	HMDB0002035
16	Formylanthranilic acid	0.039236	1.81	0.41	0.8324	HMDB0004089
	**Positive ion mode**					
1	Stearoylcarnitine	4.33E-03	2.30	1.67	0.9981	HMDB0000848
2	L-Palmitoylcarnitine	7.96E-03	2.09	1.63	0.9952	HMDB0240774
3	Oxyquinoline	1.14E-02	2.05	1.58	0.8735	--
4	β-Homoproline	1.81E-02	1.75	0.58	0.9616	HMDB0247562
5	Pargyline	2.12E-02	1.73	0.55	0.8203	HMDB0015563
6	L-Pipecolic acid	2.32E-02	1.93	0.51	0.9989	HMDB0000716
7	N-Acetyl-L-Histidine	2.58E-02	2.05	2.04	0.9958	HMDB0032055
8	Linoleoyl ethanolamide	3.04E-02	2.03	1.84	0.9704	HMDB0012252
9	N-alpha-Acetyl-L-lysine	4.28E-02	1.71	1.65	0.9252	HMDB0000446

Table S4 The primary differential metabolites related to lipid metabolism.

No.	Metabolite	Ionization mode	hmdbID
	**Mod-Con**		
1	all cis-(6,9,12)-Linolenic acid	**NEG**	HMDB0003073
2	Linoleic acid	**NEG**	HMDB0000673
3	7,10,13,16-Docosatetraenoic acid	**NEG**	HMDB0002226
4	Phosphorylcholine	**NEG**	HMDB0001565
5	Glycerol 3-phosphate	**NEG**	HMDB0000126
6	Arachidonic Acid	**NEG**	HMDB0001043
7	1-Palmitoyl Lysophosphatidic Acid	**NEG**	HMDB0010382
8	12R-HETE	**NEG**	HMDB0062290
9	PC(16:0/16:0)	**POS**	HMDB0000564
10	Glycerophosphocholine	**POS**	HMDB0000086
11	20-Hydroxyarachidonic acid	**POS**	HMDB0005998
	**HD-Mod**		
1	Myristic acid	**NEG**	HMDB0000806
2	cis-9-Palmitoleic acid	**NEG**	HMDB0003229
3	Docosahexaenoic acid	**NEG**	HMDB0002183
4	1-Palmitoyl Lysophosphatidic Acid	**NEG**	HMDB0007855
5	Stearoylcarnitine	**POS**	HMDB0000848
6	L-Palmitoylcarnitine	**POS**	HMDB0240774
7	1-Palmitoyllysophosphatidylcholine	**POS**	HMDB0010382
8	Palmitoyl ethanolamide	**POS**	HMDB0002100

Highlighted in red were the common lipid-related differential metabolites between HD-Mod and posCon-Mod.
